# Molecular and Ultrastructural Mechanisms Underlying Yellow Dwarf Symptom Formation in Wheat after Infection of Barley Yellow Dwarf Virus

**DOI:** 10.3390/ijms19041187

**Published:** 2018-04-13

**Authors:** Wei Rong, Xindong Wang, Xifeng Wang, Sebastien Massart, Zengyan Zhang

**Affiliations:** 1 Institute of Crop Sciences, National Key Facility for Crop Gene Resources and Genetic Improvement, Chinese Academy of Agricultural Sciences, Beijing 100081, China; 18931648844sabrina@gmail.com (W.R.); xidongwang@163.com (X.W.); 2 Laboratory of Integrated and Urban Phytopathology, Gembloux Agro-Bio Tech-University of Liège, Passage des déportés, 2, 5030 Gembloux, Belgium; 3 Institute of Plant Protection, Chinese Academy of Agricultural Sciences, Beijing 100193, China; xfwang@ippcaas.cn

**Keywords:** *Tritium aestivum* L., barley yellow dwarf virus, yellow dwarf symptom formation, chloroplast, chlorophyll, abscisic acid, ethylene, reactive oxygen species

## Abstract

Wheat (*Tritium aestivum* L.) production is essential for global food security. Infection of barley yellow dwarf virus-GAV (BYDV-GAV) results in wheat showing leaf yellowing and plant dwarfism symptom. To explore the molecular and ultrastructural mechanisms underlying yellow dwarf symptom formation in BYDV-GAV-infected wheat, we investigated the chloroplast ultrastructure via transmission electron microscopy (TEM), examined the contents of the virus, H_2_O_2_, and chlorophyll in Zhong8601, and studied the comparative transcriptome through microarray analyses in the susceptible wheat line Zhong8601 after virus infection. TEM images indicated that chloroplasts in BYDV-GAV-infected Zhong8601 leaf cells were fragmentized. Where thylakoids were not well developed, starch granules and plastoglobules were rare. Compared with mock-inoculated Zhong8601, chlorophyll content was markedly reduced, but the virus and H_2_O_2_ contents were significantly higher in BYDV-GAV-infected Zhong8601. The transcriptomic analyses revealed that chlorophyll biosynthesis and chloroplast related transcripts, encoding chlorophyll a/b binding protein, glucose-6-phosphate/phosphate translocator 2, and glutamyl-tRNA reductase 1, were down-regulated in BYDV-GAV-infected Zhong8601. Some phytohormone signaling-related transcripts, including abscisic acid (ABA) signaling factors (phospholipase D alpha 1 and calcineurin B-like protein 9) and nine ethylene response factors, were up-regulated. Additionally, reactive oxygen species (ROS)-related genes were transcriptionally regulated in BYDV-GAV infected Zhong8601, including three up-regulated transcripts encoding germin-like proteins (promoting ROS accumulation) and four down-regulated transcripts encoding peroxides (scavenging ROS). These results clearly suggest that the yellow dwarf symptom formation is mainly attributed to reduced chlorophyll content and fragmentized chloroplasts caused by down-regulation of the chlorophyll and chloroplast biosynthesis related genes, ROS excessive accumulation, and precisely transcriptional regulation of the above-mentioned ABA and ethylene signaling- and ROS-related genes in susceptible wheat infected by BYDV-GAV.

## 1. Introduction

Barley yellow dwarf viruses (BYDVs) are a group of related single-stranded RNA viruses assigned to the genus *Luteovirus* or *Polerovrus* or unassigned to a genus [1,2]. They are transmitted by several cereal aphid species in a persistent and circulative-propagative manner [1]. Based on the specificity of their aphid vectors and sequences of the viruses, BYDVs have been divided into ten distinct species, BYDV-PAV, PAS, MAV, kerII and kerIII in the genus *Luteovirus*, cereal yellow dwarf virus RPV, RPS, maize yellow dwarf virus RMV in the genus *Polerovirus*, BYDV-GPV, and SGV as unassigned members in family *Luteoviridae* [2,3,4,5]. BYDV-GAV, which is the most popular species found in China in recently years, is a Chinese isolate of BYDV-MAV [5]. BYDVs can infect most important cereal crops and grass species of the family *Gramineae* (*Poaceae*), such as wheat (*Triticum aestivum*), barley (*Hordeum vulgare*), and oat (*Avena sativa*) [6,7]. Wheat is one of the most important food crops around the world, and its production is essential for global food security. Infection of BYDVs results in wheat showing the symptoms of leaf yellowing and plant dwarfism, ultimately leading to yield loss [7,8,9].

No complete resistance to BYDVs has been identified in wheat. Several wild relatives of wheat, such as *Thinopyrum intermedium* (*Th. intermedium*), harbor resistance genes against BYDVs. Certain alien chromosomal fragments with BYDV resistance genes have been introgressed into wheat and resistant wheat-alien translocation lines were generated [7,8,9,10], as well as molecular markers for monitoring these resistance chromosomal fragments were developed [11,12]. Recently, the resistant wheat-*Th. intermedium* translocation plants at seedling stage and comparative transcriptome analyses were deployed to identify resistance-related genes from the translocation line [13]. Nevertheless, mechanisms underlying the yellow dwarf symptom formation in BYDVs-susceptible wheat cultivars have not been reported yet.

In other plant species, several papers explored the mechanisms underlying leaf yellowing symptom caused by different virus infections [14,15]. For instance, in rice stripe virus–infected rice, the down-regulation of certain chlorophyll biosynthesis genes, such as magnesium chelatase subunit I (CHLI) and subunit D (CHLD), can result in chlorophyll reduction and leaf chlorosis [15]. In African cassava mosaic virus (ACMV)-infected cassava, the leaf yellowing symptom formation might be owed to chlorophyll degradation and dropped expressional levels of genes encoding the major apoproteins in light-harvesting complex II [16]. In previous papers, it was reported that in susceptible *Prunus* plants infected by plum pox virus, oxidative stress due to accumulation of hydrogen peroxide (H_2_O_2_) and an imbalance in the antioxidative systems played a role in the disease symptom formation [17,18]. In pea plants infected by plum pox virus, the alteration in the chloroplastic metabolism was produced, leading to the accumulation of reactive oxygen species (ROS) in this cell organelle and leaf yellowing [19]. Previous studies also showed that plant height is regulated by certain phytohormones, such as gibberellin acid (GA), abscisic acid (ABA), and ethylene (ET) [20]. The alternation of phytohormone biosynthesis/signaling related transcripts could significantly influence the developmental processes in plants [21]. Currently, little is known about which and how genes contribute to the symptoms on wheat induced by invasion of BYDVs.

In this report, to unravel basal causes of the yellowing dwarf symptom formation in susceptible wheat induced by BYDV-GAV, we observed the host chloroplasts by transmission electron microscopy (TEM), studied the comparative transcriptome, and examined contents of the virus, H_2_O_2_, and chlorophyll in the susceptible wheat line Zhong8601 after BYDV-GAV inoculation.

## 2. Results

### 2.1. BYDV-GAV and Chlorophyll Contents Were Changed after BYDV-GAV Inoculated Zhong8601

**Figure 1:**
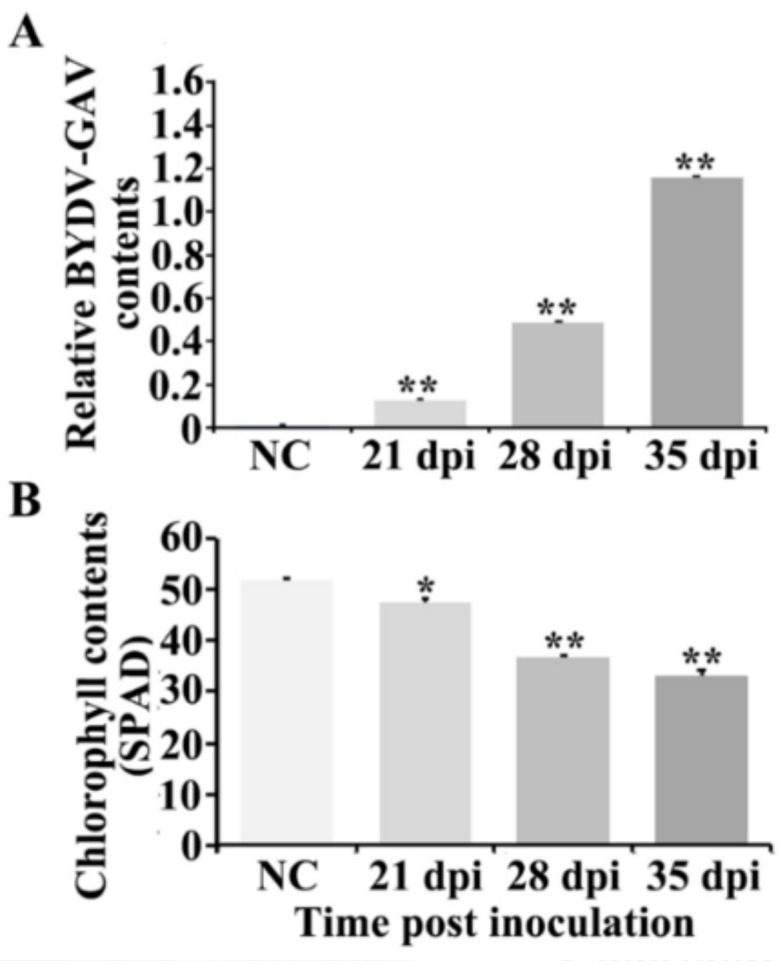
Relative virus and chlorophyll contents in barley yellow dwarf virus-GAV (BYDV-GAV)-inoculated Zhong8601. (**A**) Enzyme-linked immunosorbent assay (ELISA) data in BYDV-GAV inoculated Zhong8601 for 21, 28 and 35 dpi, and non-inoculated Zhong8601 (NC, control); (**B**) Chlorophyll contents in BYDV-GAV inoculated Zhong8601 for 21, 28 and 35 dpi, and non-inoculated Zhong8601 (NC, control). Statistically significant differences were determined using Student’s *t*-test (* *p* < 0.05; ** *p* < 0.01, statistically significant difference). Three technological repeated data of each sample pool (each sample pool was a mixture of ten individual leaves collected at each tested time point) were analyzed, respectively.

Enzyme-linked immunosorbent assay (ELISA) was used to identify the relative BYDV-GAV contents in leaves of BYDV-GAV inoculated Zhong8601 for 21, 28 and 35 dpi, and of non-inoculated Zhong8601 (NC, control). As shown in Figure 1A, the accumulations of BYDV-GAV titers increased with BYDV-GAV inoculation time. Judging from the results, BYDV-GAV titers in BYDV-susceptible Zhong8601 were markedly elevated after the virus infection, and the ELISA value in BYDV-GAV inoculated Zhong8601 at 35 dpi was 1.158 ± 0.007 and about 165.43-fold higher than that in non-inoculated Zhong8601 (0.007 ± 0.002) (Figure 1A). The ELISA value 0.1 was set as a threshold for virus-infected plants with susceptibility according to earlier reports [22]. The data indicated that after the virus inoculation, the BYDV-GAV was successfully infected in BYDV-susceptible Zhong8601 and could replicated in the infected Zhong8601 plants. The ELISA data also confirmed that Zhong8601 plants were infected after inoculation with BYDV-GAV.

We further examined the chlorophyll contents in leaves of Zhong8601 at 21, 28 and 35 dpi with BYDV-GAV and of non-inoculated Zhong8601 (NC, control). The chlorophyll contents in the BYDV-GAV-inoculated leaves were significantly lower than that in non-inoculated Zhong8601 (Figure 1B). The chlorophyll contents in BYDV-GAV inoculated Zhong8601 leaves were 46.56 ± 0.075 SPAD at 21 dpi, 36.18 ± 0.030 SPAD at 28 dpi, and 32.61 ± 0.83 SPAD at 35 dpi, respectively, whereas chlorophyll content was 50.98 ± 0.15 SPAD in non-inoculated Zhong8601. The results indicated that the chlorophyll contents were significantly decreased in susceptible wheat after BYDV inoculation.

### 2.2. The Chloroplasts in Zhong8601 Were Influenced after BYDV Inoculation

**Figure 2:**
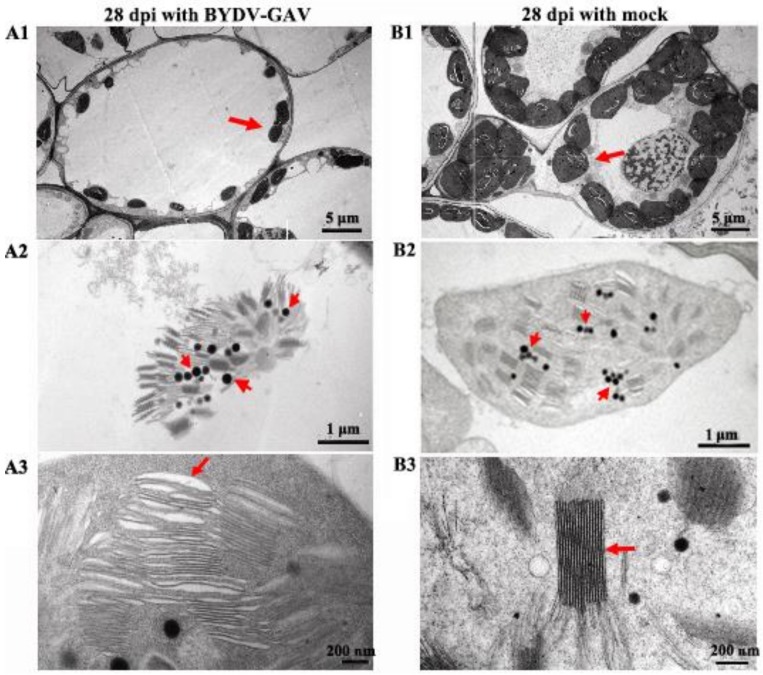
Transmission electron microscopy (TEM) observation on Zhong8601 after BYDV-GAV inoculation (**A**) and mock inoculation (**B**) for 28 d. (**A1**,**B1**) comparing the number of chloroplasts in one cell between in BYDV-GAV and mock inoculated Zhong8601. Each arrow is pointing at one chloroplast, respectively; (**A2**,**B2**) comparing integrity of chloroplasts between in BYDV-GAV and mock inoculated Zhong8601. The arrows indicate grana; (**A3**,**B3**) comparing densities of thylakoids in chloroplasts between BYDV-GAV and mock inoculated Zhong8601. Arrow indicates the thylakoid.

In the nursery field, the yellowing symptom could be observed from the leaves of susceptible wheat line Zhong8601 infected with BYDV-GAV as early as 28 dpi [13]. In order to explore the relationship between chloroplasts and the leaf yellowing symptom in BYDV-GAV-infected Zhong8601, the chloroplasts from leaf cells of Zhong8601 at 28 dpi were observed by TEM. TEM results showed that compared to mock-inoculated Zhong8601 (Figure 2(B1–B3)), the number of chloroplasts was reduced, and the chloroplasts in the cells of BYDV-GAV infected Zhong8601 were no longer intact or did not possess the normal pattern (Figure 2(A1–A3)). In these chloroplasts in BYDV-GAV-infected Zhong8601, the thylakoids were not well developed (Figure 2(A3)). Furthermore, the grana in some chloroplasts were unsuccessfully formed, and the plastoglobules or starch granules could barely be observed in the chloroplasts of BYDV-GAV-infected Zhong8601 (Figure 2(A2,A3)). These results could suggest that BYDV-GAV infection negatively influenced the normal pattern and integrality of the chloroplasts in susceptible wheat.

### 2.3. Global Transcripts Were Altered in BYDV-Infected Zhong8601 through Comparative Transcriptome 

**Figure 3:**
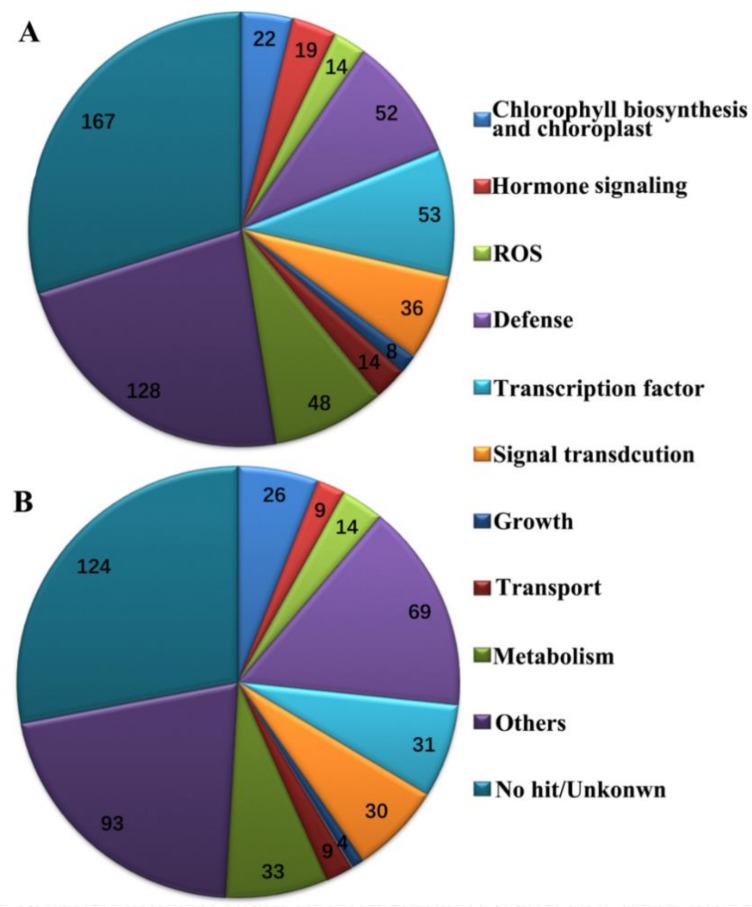
Functional categories of differentially expressed transcripts in BYDV-GAV-inoculated Zhong8601 for 35 dpi relative to mock-inoculated Zhong8601 for 35 dpi. (**A**) Pathway enrichments of up-regulated transcripts; (**B**) Pathway enrichments of down-regulated transcripts.

In the nursery field, the typical BYDV symptoms—leaf yellowing and plant dwarfism—obviously occur in the spring susceptible wheat plants at 35 dpi with BYDV-GAV. Thus, the RNAs from BYDV-GAV-inoculated Zhong8601 for 35 dpi (SI35) and mock-inoculated Zhong8601 (SM35) were subjected to microarray analysis. By means of bioinformatic analysis, we identified 1031 BYDV-responsive genes in susceptible Zhong8601 between BYDV-GAV and mock infected leaves for 35 dpi (Table S1). In Zhong8601 leaves inoculated with BYDV-GAV for 35 dpi, 561 up- and 442 down-regulated transcripts were identified after removing the duplications (Table S2). We further analyzed the differential expressed transcripts between the BYDV-GAV-inoculated Zhong8601 and mock-inoculated Zhong8601 in order to explore molecular mechanisms underlying the yellowing dwarf symptom formation in susceptible wheat with BYDV inoculation. According to the Gene Ontology and category methods as previously described [23,24,25], these differentially transcriptional genes were divided into 11 categories, including chlorophyll biosynthesis and chloroplast, hormone signaling, ROS, defense, transcription factor, signal transduction, growth, transport, metabolism, others, and no hit/unknown (Figure 3; Table S2).

Of them, 52 up-regulated and 69 down-regulated defense related transcripts were also found in Zhong8601 at 35 dpi with BYDV-GAV compared to mock inoculation (Table S2). These 52 up-regulated transcripts mainly included seven lipid transfer proteins (LTPs), six disease-resistance proteins, six heat shock protein, three mitochondrial outer membrane porins, and 30 other transcripts encoding additional plant defense-related proteins. The 69 down-regulated defense related transcripts included seven chitinases (Chits), six metallothioneine type 2, four proline-rich protein, four glucan 1,3-β-glucosidase, three beta-glucosidase, three disease resistance protein, three lipid transfer proteins (LTPs), three lipoxygenases, and 36 other transcripts encoding other plant defense-related proteins. It can be seen from here that these defense-related proteins encoded by the up-regulated and down-regulated transcripts were varied with each other. Meanwhile, 53 up-regulated and 31 down-regulated transcription factors and 39 up-regulated and 30 down-regulated transcripts involved in signal transduction were also identified from the microarray data at 35 dpi with BYDV-GAV, respectively (Figure 3; Table S2). The 53 up-regulated transcription factors included seven zinc finger proteins, seven transcription regulators, and 39 other transcription factors. The 31 down-regulated transcription factors included transcripts encoding four ABC transports, four histone proteins, three MYB transcription factors, and 20 other transcription factors. The 39 up-regulated transcripts involved in signal transduction were 20 protein kinases, four calcium-binding proteins, three C2 domain containing proteins, and 12 other transcripts related to signal transduction, while the 30 down-regulated transcripts involved in signal transduction were eight protein kinases, four dirigent proteins, and 18 other transcripts related to signal transduction. Both the transcription factors and signal transduction encoding transcripts were also different with each other in the up-regulated and down-regulated transcripts (Table S2). These data suggested that these genes were precisely regulated in wheat upon BYDV-GAV-inoculation and might play roles in basal resistance and necessary growth of wheat plants.

Hereafter, we will focus on investigating these differentially expressing transcripts involving in chlorophyll biosynthesis and chloroplast, phytohormone biosynthesis/signaling, and ROS, which might be mainly responsible for the yellow dwarf symptom in Zhong8601.

### 2.4. Chlorophyll Biosynthesis and Chloroplast Related Transcripts Were Down-Regulated after BYDV Infection

Through analyzing the transcriptome data, 48 chlorophyll- and chloroplast-related transcripts were significantly differentially regulated upon BYDV-GAV inoculation for 35 dpi (Table S3). Among them, 22 up-regulated and 26 down-regulated chlorophyll and chloroplast related transcripts were identified through SI35 vs. SM35 pairwise comparison (Table S3). The expressional levels of nine down-regulated chlorophyll biosynthesis and chloroplast related transcripts were markedly lower (2.51- to 1017.08-fold) in BYDV-GAV inoculated Zhong8601 leaves than in mock inoculated Zhong8601 leaves. These nine chlorophyll biosynthesis– and chloroplast-related transcripts included seven encoding Cab proteins, one encoding glucose-6-phosphate/phosphate translocator 2 (GPT2), and one encoding glutamyl-tRNA reductase 1 (GluTR1) (Table S3). Additionally, the remaining 17 down-regulated transcripts encoded starch branching enzyme I, photosystem II reaction center protein Z, photosystem II stability/assembly factor HCF136, and photosystem II light harvesting complex protein, respectively. Previous paper documented that GPT2 played a role during starch synthesis through maintaining the intactness of chloroplasts [26]. In *Arabidopsis thaliana*, partial silencing of *GluTR1* can lead to chloroplast deficiency ranging from partial yellow to total yellow color of the leaf [27].

### 2.5. Phytohormone Signaling Related Transcripts Were Differentially Regulated in BYDV-Infected Zhong8601

The comparative transcriptome analyses showed that 28 transcripts related to phytohormone biosynthesis/signaling pathways were differentially regulated upon BYDV-GAV inoculation for 35 dpi (Table S4). Among them, 20 up-regulated and eight down-regulated transcripts uniquely detected from BYDV-infected leaves at 35 dpi were related to phytohormone pathway (Table S4). Of these 28 phytohormone pathway related transcripts at 35 dpi with BYDV-GAV, nine ET signaling related transcripts and four ABA signaling related transcripts could be found only from the up-regulated transcripts (Table S4). These nine ET signaling related transcripts encode two AP2 domain-containing proteins (AP2), one AP2/ERF domain-containing protein in B3 subgroup (AP2/ERF-B3), three ethylene-responsive element binding protein (EREBP), and three ethylene-responsive factors (ERF), respectively. Of the four up-regulated transcripts associated with ABA biosynthesis, three encoded phospholipase D alpha 1 protein (PLD1) homologs and one encoded calcineurin B-like protein 9 (CBL9) homolog. *PLD1* has been proved to mediate the ABA transduction in barley aleurone [28]. *CBL9* has been reported to play a role in ABA signaling pathway in *Arabidopsis* [29]. Comparing with the mock inoculated Zhong8601, the transcriptional levels of these transcripts were 4.67- to 309.50-fold higher in BYDV-GAV inoculated Zhong8601 at 35 dpi (Table S4).

Apart from these transcripts, three up-regulated and three down-regulated jasmonate (JA) signaling related transcripts, and two up-regulated and one down-regulated GA signaling related transcripts were also identified, respectively. Among the up-regulated and down-regulated JA signaling related transcripts, transcripts could encode 12-oxophytodienoate acid reductase (OPR). *OPR* has been reported as involving in JA biosynthesis in many plants including wheat [30]. The GA singling pathway-related transcripts included one up-regulated gibberellin receptor GID1L2, one up-regulated gibberellin stimulated transcript, and one down-regulated gibberellin receptor GID1L3, respectively. In vascular plants, these three genes all could participate in the GA-GID1-DELLA model and regulated GA signaling cascade [31]. Through interacting with second messengers and other plant hormones, like JA, these transcripts could mediate diverse growth and developmental process through the life cycle of plants [31]. Even though the transcripts related to both JA and GA signaling pathways did not have a consistent regulation tendency as ABA or ET, the antagonism between JA-GA signaling pathways has been reported [32,33].

### 2.6. ROS-Related Transcripts and H_2_O_2_ Accumulation also Participated in BYDV Symptom Formation in Zhong8601

The comparative transcriptome analyses revealed that 14 up-regulated and 14 down-regulated transcripts were detected as ROS-related genes in BYDV-GAV inoculated Zhong8601 compared to mock inoculated Zhong8601 for 35 dpi (Table S5). Among these transcripts, three kinds of transcripts included glutathione S-transferase (GST), germin-like protein, and peroxidase (POX), which have been well reported about their roles in ROS metabolism in plants. By analyzing the microarray data in Zhong8601 at 35 dpi with BYDV-GAV and mock, eight up-regulated transcripts encoding three GSTs, three germin-like proteins, and two POXs, respectively, and seven down-regulated transcripts encoding three GSTs and four POXs, respectively, were identified (Table S5). According to previous reports, GSTs can alter the anti-oxidant metabolism in plants, germin-like proteins play roles during the promotion of ROS accumulation in plants, and the POXs were mainly in charge of ROS scavenging [34,35,36]. The data suggested that transcriptional levels of three germin-like proteins were increased and two POX transcripts were decreased in BYDV-GAV inoculated Zhong8601 leaves compared to mock inoculated Zhong8601 leaves, implying that ROS content might be elevated in BYDV-GAV inoculated Zhong8601 leaves.

**Figure 4:**
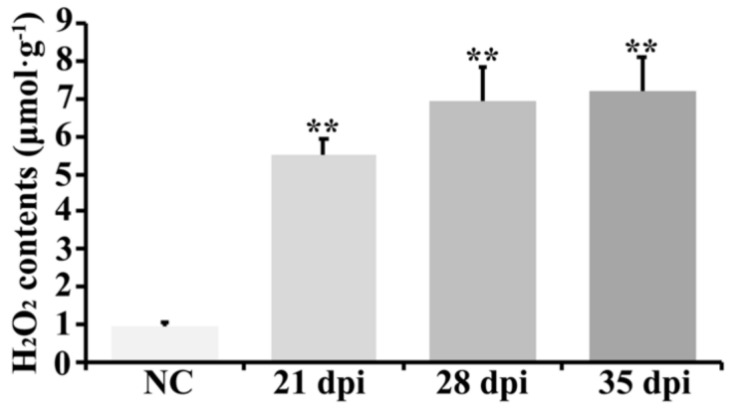
H_2_O_2_ contents in BYDV-GAV inoculated Zhong8601. H_2_O_2_ contents in BYDV-GAV-inoculated Zhong8601 for 21, 28 and 35 dpi, and non-inoculated Zhong8601 (NC, control). Statistically significant differences were determined by using Student’s *t*-test (** *p* < 0.01, statistically significant difference). Three technological repeated data of each sample pool (the sample pool used were mixtures of ten individual leaves collected at each tested time point) were analyzed respectively.

To explore if the expression of the above ROS-related genes affects H_2_O_2_ contents in BYDV-GAV infected Zhong8601, we examined the H_2_O_2_ contents in the newly emerged leaves from both BYDV-GAV and non-inoculated Zhong8601 for 21, 28, and 35 dpi, meanwhile non-inoculated Zhong8601 was used as control. The results indicated that in the leaves of BYDV-susceptible Zhong8601, the H_2_O_2_ concentrations were remarkably increased during the tested time points in the BYDV-GAV inoculated Zhong8601. As shown in Figure 4, H_2_O_2_ concentrations in the BYDV-GAV inoculated Zhong8601 were 5.53 ± 0.41 μmol·g^−1^ at 21 dpi, 6.93 ± 0.92 μmol·g^−1^ at 28 dpi, and 7.21 ± 0.89 μmol·g^−1^ at 35 dpi, respectively, while H_2_O_2_ concentrations in non-inoculated Zhong8601 was 0.94 ± 0.12 μmol·g^−1^. The data indicated that ROS indeed was accumulated in BYDV-infected Zhong8601, demonstrating ROS elevated inference from transcriptomic analysis results. The results suggested that ROS accumulation also might be involved in the BYDV symptom formation induced by BYDV infection in susceptible wheat.

### 2.7. The Microarray Data Confirmation by RT-qPCR

**Figure 5:**
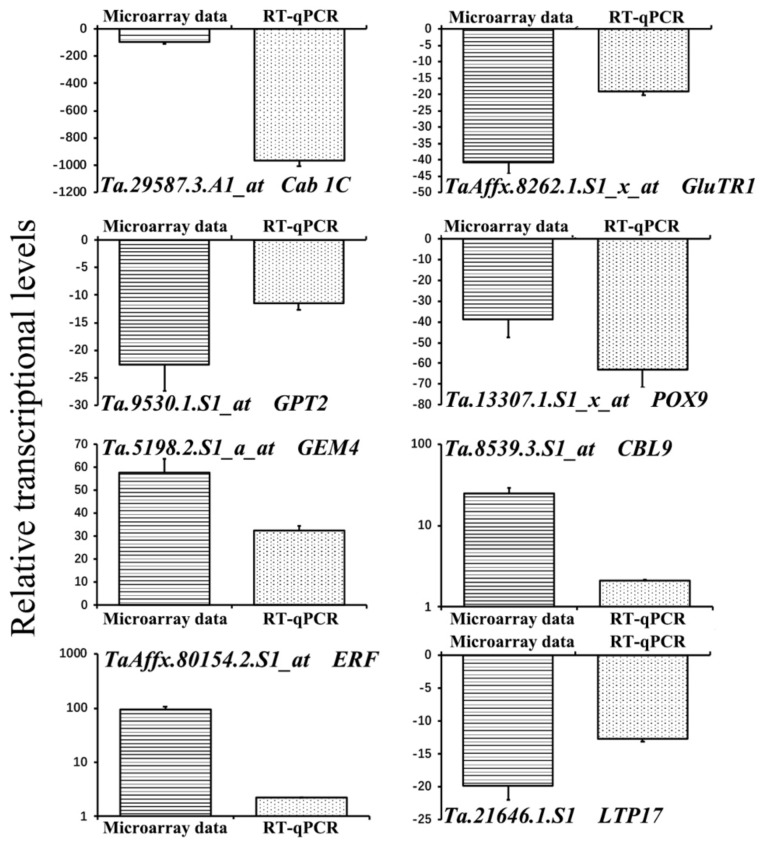
Comparison of data between microarray and RT-qPCR analyses on transcripts related to BYDV symptom formation in BYDV-GAV inoculated Zhong8601 at 35 dpi compared to mock inoculated Zhong8601. Three technical replicates were averaged for microarray data and RT-qPCR analysis, respectively. Bars indicate standard error of the mean (SE). The samples used were mixtures of the ten individual leaves as a sample pool collected at 35 dpi.

The transcriptional levels of three down-regulated chlorophyll biosynthesis and chloroplasts related transcripts, two ROS-related transcripts, two up-regulated phytohormone pathway–related transcripts, and one down-regulated plant defense-related transcript were investigated by real-time quantitative PCR (RT-qPCR). The three down-regulated chlorophyll biosynthesis and chloroplasts were Ta.29587.3.A1_at, *Cab 1C*; TaAffx.8262.1.S1_x_at, *GluTR1*; and Ta9530.1.S1_at, GPT2. The two ROS-related transcripts included one down-regulated transcripts Ta.13307.1.S1_x_at, *POX9* and one up-regulated transcript Ta.5198.2.S1_a_at, *GEM4*, and the two up-regulated phytohormone pathway–related transcripts were Ta.8539.3.S1_at, *CBL9* and TaAffx.80154.2.S1_at, *ERF*, and the one down-regulated plant defense-related transcript was Ta.21646.1.S1, *LTP17*, respectively. As shown in Figure 5, the RT-qPCR analysis results were consistent with the microarray data tendency. These results indicated that the results of microarray analyses were convinced.

## 3. Discussion

The present study combines microarray-based transcriptome, TEM, and chlorophyll-level analyses to explore the mechanisms involved in the yellowing and dwarfism symptom development caused by infection by BYDV in the susceptible wheat line Zhong8601. These results revealed that after BYDV-GAV infection in the susceptible wheat, drop expressions of chlorophyll biosynthesis and chloroplast related genes, and altered expression of the above-mentioned ABA and ET signaling- and ROS-related genes might contribute to the lower chlorophyll content and fragmentized chloroplasts, ROS accumulation and slower growth, consequently resulting in leaf yellowing and plant dwarfing. To our knowledge, this is the first study on mechanisms underlying the symptom formation in BYDV-GAV susceptible wheat.

### 3.1. Major Mechanism for Yellowing Symptom Formation after BYDV-GAV Inoculation

Cab protein has been demonstrated to be responsible for chlorophyll accumulation and starch synthesis [37]. Our transcriptome analyses indicated that seven *Cab* encoding transcripts, involving in chlorophyll biosynthesis, were down-regulated in Zhong8601 after BYDV-GAV infection for 35 d. It was reported that chlorophyll biosynthesis enzymes could be independently regulated, and inhibition of each step might affect the chlorophyll biosynthesis and results in leaf color change [38]. Here, apart from the seven transcripts corresponding to the mentioned above Cabs, two additional chloroplast related transcripts, encoding GPT2, and one GluTR1, were down-regulated at 35 dpi with BYDV-GAV. GPT2 plays an important role in starch synthesis and in maintaining the intactness of chloroplasts [26]. Previous study reported that partially silencing of GluTR1 in *Arabidopsis* could result in chloroplast deficiency, ranging from partial yellow to total yellow [27]. Moreover, at 35 dpi with BYDV-GAV, the expression levels of the genes, corresponding to starch branching enzyme I, photosystem II reaction center protein Z, photosystem II stability/assembly factor HCF136, and photosystem II light harvesting complex protein, were decreased. These results suggest that after BYDV-GAV infection, the decreased expression of these chlorophyll biosynthesis and chloroplast related genes may lead to deficiency in chlorophyll biosynthesis and chloroplasts in Zhong8601. In fact, chlorophyll contents in BYDV-GAV infected Zhong8601 indeed are markedly decreased. Taking our data of chlorophyll reduction in virus-infected leaves with previous finding in tobacco mosaic virus (TMV)-infected leaf yellowing associated with decreased chlorophyll contents [23], these results support the notion that leaf yellowing is closely associated with chlorophyll reduction. Furthermore, the TEM observation indicated that in leaves of Zhong8601 after BYDV-GAV inoculation for 28 dpi, there were small and cracked chloroplasts, where thylakoids, starches and plastoglobules could not be well developed. These results clearly reveal that after BYDV-GAV infection, the susceptible wheat line Zhong8601 cannot maintain intact chloroplasts and fail to operate high efficiency of chlorophyll biosynthesis. In turn, the decrease of chlorophyll accumulation and starch synthesis even chloroplast deficiency resulted in the leaf yellowing.

Previous papers documented that after virus infection in susceptible *Prunus* species, the chloroplastic metabolism was altered and ROS was accumulated, resulting in leaf yellowing [17,18,19]. In the current research, the microarray data of ROS-related transcripts showed that both the up- and down-regulated transcripts involved with ROS accumulation and scavenging were also identified in BYDV-inoculated Zhong8601. Of them, four out of six transcripts encoding peroxidase, which is in charge of ROS scavenging, were down-regulated (Table S5). Meanwhile, three germin-like proteins, which could positively promote ROS accumulation in plants, were only detected as up-regulated (Table S5). With the down-regulation of ROS scavenging transcripts and the up-regulation of ROS accumulation promoting transcripts, the ROS balance in damaged chloroplasts of Zhong8601 was also broken and ROS was accumulated after BYDV-GAV infection. The H_2_O_2_ assay results supported the notion that H_2_O_2_ concentration was remarkably increased in BYDV-GAV infected Zhong8601 and revealed that ROS excessive accumulation might be one cause for leaf yellowing of susceptible wheat infected by BYDV-GAV. Our results were consistent with the previous studies [19].

Furthermore, ABA and ET have been proven to be senescence-promoting [24,25]. Recent studies showed that exogenous application of ET could accelerate leaf yellowing [39], and genes involving in ABA biosynthesis/signaling were up-regulated during leave yellowing upon abiotic and biotic stresses [25]. Based on the transcriptome data at 35 dpi, we deduced that the elevated expression of the ABA and ET signaling related genes might be involved in the yellowing symptom detected on the BYDV-GAV inoculated wheat plants.

### 3.2. Major Cause for Plant Dwarfism Formation after BYDV Inoculation

In plants, the varied transcriptional levels of genes in the biosynthesis/signaling pathways of phytohormones (ABA, ET, JA, and GA) have been found to influence plant vascular tissue differentiations, resulting in modification of plant heights/plant dwarfism [20,40,41]. Based on 35 dpi transcriptome data, nine ET signaling related transcripts (i.e., two *AP2*, one *AP2/ERF-B3*, three *EREBP*, and three *ERF*) and four ABA signaling related transcripts (three *PLD1* and one *CBL9*) were up-regulated, respectively. Recent studies indicated that overexpression of ET/ABA signaling related genes at late plant-pathogen interaction stage could lead to plant dwarfism in order to maintain a balance between plant growth and stress defense [40,41]. In tomato (*Solanum lycopersicum* L.), *Soly19* and *Soly66*, encoding AP2/ERF-B3 containing proteins, were up-regulated upon Tomato yellow leaf curly virus (TYLCV) infection in the TYLCV-susceptible tomato varieties and contributed to the dwarfism phenotype after TYLCV infection [40]. Up-regulation of *AtERF6* in *Arabidopsis* upon stresses could also lead to plant dwarfism eventually [41]. Moreover, *PLD1* and *CBL9* were up-regulated in wheat after BYDV-GAV infection, respectively. *PLD1* and *CBL9* have been previously proved to participate in ABA biosynthesis/signaling [28,29,42]. Thus, up-regulation of nine ET and four ABA signaling related transcripts in susceptible wheat at the late BYDV-GAV inoculation stage should partially contribute to the dwarfism phenotype observed.

It has been known that during the whole plant growth cycle, plants defense upon pathogens usually at the expense of growth, and the defense/growth trade-off is a dynamic process that is indispensable for plants [32]. Among this dynamic process, GA regulates many essential plant developmental processes, while JA plays a dominant role in mediating plant response to necrotrophic pathogens [32]. Even though the transcriptional levels of JA and GA do not have a unified transcriptional tendency after BYDV-GAV inoculation for 35 dpi in our study, the knowledge that the intensive crosstalk between these two phytohormones and their parts in orchestrate growth and defense responses to stresses in plants still aroused our attention. With both the up- and down-regulation of *OPR*, which is related to JA biosynthesis in many plants including wheat [30], and the two up-regulated transcripts (gibberellin receptor GID1L2 and gibberellin stimulated transcripts) and one down-regulated transcript encoding gibberellin receptor GID1L3, all which are the important players in regulating GA signaling cascade in vascular plant [31]. According to the varied transcriptional levels of both JA and GA signaling pathways related transcripts in Zhong8601 upon BYDV-GAV inoculation for 35 dpi, it could be induced that both JA and GA signaling pathways were influenced by BYDV-GAV infection. These results suggested that after BYDV-GAV inoculation, the altered expression levels of these genes in these phytohormones’ pathway in the susceptible Zhong8601 plants might result in the plant dwarfism.

Additionally, it had been reported that the drop of chlorophyll content can limit plant height and grain weight in wheat [43]. We deduced that the reduced chlorophyll content and ROS excessive accumulation in BYDV-GAV inoculated might partially contribute to plant dwarfism and yield reduction in susceptible wheat induced by infection of BYDV-GAV.

## 4. Materials and Methods

### 4.1. Plant Materials, BYDV-GAVIsolate, and Treatments

The BYDV-susceptible spring wheat line Zhong8601 was maintained in the laboratory of Zengyan Zhang, Institute of Crop Science, Chinese Academy of Agricultural Sciences (CAAS), Beijing, China. The BYDV-GAV was provided by Yan Liu, Institute of Plant Protection, CAAS, China.

Three-hundred Zhong8601 plants were planted in the nursery in February for two years successively. The trial was designed with 15 rows, the length of each row is 1.2 m and the distance between two rows is 25 cm. At their two- or three-leaf stages, 200 plants were inoculated with viruliferous aphids carrying BYDV-GAV, and the other 100 plants were inoculated with non-viruliferous aphids as control (mock) at the same time, 10 aphids per plant. Plastic covers were used to proof cross contamination.

### 4.2. RNA Extraction and the First-Strand cDNA Synthesis

Total RNAs were extracted from leaf tissues of Zhong8601 after BYDV-GAV and mock inoculation using Trizol reagent (Invitrogen, Carlsbad, CA, USA), and subjected to RNase-free DNase for digestion and purification. Five micrograms (μg) RNA of each sample was used to synthesize first-strand cDNA using the Superscript II First-Strand Synthesis Kit for RT-qPCR (Invitrogen).

### 4.3. Virus Diagnostics by ELISA in Zhong8601 Leaves with BYDV-GAV and Mock Inoculation

The Zhong8601 leaf sections near the bitten sites of aphids carried with BYDV-GAV, or the sections with BYDV symptoms were sampled from ten individual plants at each tested time point (i.e., 21, 28 and 35 dpi). Each treatment was conducted with three technical replicates (each sample at each tested time point was pool mixed with ten individual leaves collected at each tested time point). ELISA were used to examine the virus titers of the collected samples. The virus titer of samples without treatment were used as control. This experiment was carried out according to the protocol of BYDV ELISA Reagent Set (SRA26500, Agdia, Elkhart, IN, USA). The absorbance was measured at 405 nm within 30 min. The ELISA value for each tested time point was an average of the three-technical replicate. Statistical analysis via Student’s *t*-test was used to compare the difference of ELISA data between BYDV-GAV inoculated Zhong8601 and non-inoculated Zhong8601.

### 4.4. Examination of Chlorophyll and H_2_O_2_ Contents in Zhong8601

Leaves of BYDV-GAV inoculated Zhong8601 for 21, 28 and 35 dpi and non-inoculated Zhong8601 (NC, control) were sampled and subjected to measure both chlorophyll contents by SPAD 502 Plus Chlorophyll Meter and to examine the H_2_O_2_ content according to a previously reported protocol [44]. Three technically repeated measuring data of each sample pool (each mixed with ten individual leaves, and the value for each measuring is the mean value of the ten biologically repeated data generated from the ten individual leaves) collected at each tested time point were analyzed respectively, and the results were the mean values from the three technically repeated data. Statistical analysis through Student’s *t*-test was used to compare differences of chlorophyll and H_2_O_2_ contents between BYDV-GAV inoculated Zhong8601 and non-inoculated Zhong8601.

### 4.5. TEM Assay for Samples of Zhong8601

After inoculated with BYDV-GAV for 28 dpi, small sections near yellowing parts of the second or third leaves from the tops of Zhong8601, 1–3 mm in length, were ditched and fixed in 4% glutaraldehyde (*v*/*v*) in 0.2 mol/L PBS (sodium phosphate buffer, pH = 7.2) for 6 to 8 h (vacuum treatment), and post-fixed in 1% OsO_4_ (Osmium(VIII) oxide) for 1 h, then fixed in 0.2 mol/L PBS (pH = 7.2) for 2 h. Dehydration was conducted in a graded ethanol series (50%, 60%, 70%, 80%, 90%, 95%, and 100%) following by acetone. Both cross cuttings and rip cuttings were conducted on the samples. Subsequently, the samples were filtrated and embedded in Spurr’s resin. Ultra-thin sections (80 nm) were prepared and mounted on copper grids for viewing under TEM (JEOL TEM-1200EX, Peabody, MA, USA) at an accelerating voltage of 60.0 to 80.0 kV [45]. The TEM assays with three biological replicates each sample were conducted, and more than five visual fields were observed for each repeat under TEM.

### 4.6. Microarray Analysis

Leaf samples (the second or third leaves from the top of each plant) of Zhong8601 at 35 dpi with BYDV-GAV or mock inoculation were collected, and 10 plants were mixed as a sample pool. Three sample pools at each tested time point were used as biological replicates in microarray data. Purified RNAs extracted from leaf sample pools of BYDV-GAV or mock inoculated Zhong8601 at 35 dpi were used to generate labeling cRNAs using the one-cycle target labeling and control reagents (Affymetrix). By following the recommended protocol, the labeled probes were hybridized to Affymetrix GeneChip^®^ wheat genome array. For each treatment, three replications were conducted. The hybridization, data acquisition and quality were performed at the ShanghaiBio Corporation (Shanghai, China) according to standard Affymetrix procedures. Significant differentially expressed transcripts were identified by ANOVA test (one-way-ANOVA) with a stringent cut off at filtered out using False Discovery Rate ≤ 0.05, corresponding to a *p*-value ≤ 0.01 between the BYDV-GAV and mock inoculated Zhong8601 at the same time point (35 dpi). The redundant probe sets were manually removed following Bolton et al. (2008) and Wang et al. (2013) [13,45].

Putative functions were assigned to the differentially expressed transcripts by HarvEST (http://harvest.ucr.edu/, Affymetrix Wheat 1 Chip version 1.58, *e* value ≤ 10^−10^) and based on the best BlastX search, the corresponding protein sequence were analyzed in non-redundant protein sequence database at the National Center for Biotechnology Information (NCBI, http://www.ncbi.nlm.nih.gov/, *e*-value ≤ 10^–10^). Gene Ontology (GO) terms [46] were obtained by querying the UniProt Protein Knowledgebase (http://www.uniprot.org/) with protein IDs from HarvEST. All differentially expressed transcripts were classified into functional categories according to Gene Ontology analysis using AgriGO (http://bioinfo.cau.edu.cn/agriGO) combined with UniProt.

### 4.7. Real-Time Quantitative PCR (RT-qPCR) Analysis

RT-qPCR technique was used to verify the expression of eight differentially expressed transcripts identified by microarray analysis. The sequences of primers specific to the tested genes are listed in Table S6. The RT-qPCR was carried out using the SYBR Green I Master Mix in a volume of 25 microliter (µL) and analyzed by an ABI 7500 RT-PCR system (Applied Biosystems, Foster City, CA, USA). The wheat *actin* gene (F: 5′-CACTGGAATGGTCAAGGCTG-3′; R: 5′-CTCCATGTCATCCCAGTTG-3′) was used as the internal control to normalize all data. The 2^−△△*C*t^ method [47] was used to evaluate the relative expression. Mock inoculated Zhong8601 from 35 dpi were used as control. All reactions were technically repeated three times, the samples used were sample pools, each of which were mixed with ten individual leaves collected at each tested time point.

## 5. Conclusions

**Figure 6:**
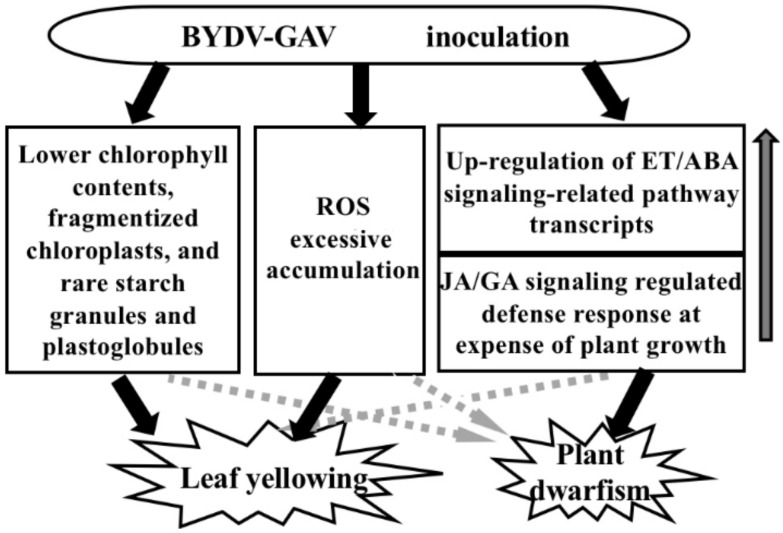
A model to explain major mechanisms underlying the yellow dwarf symptom formation in susceptible wheat induced by BYDV-GAV infection. The arrows with black lines represent the main causes responsible for the yellow dwarf symptom formation in susceptible wheat. The arrows with dashed lines represent that the reasons may also be related with the symptoms, but not be the main reasons responsible for these symptoms. ET indicates ethylene. ABA indicates abscisic acid. JA indicates jasmonate acid. GA indicates gibberellin acid. ROS indicates reactive oxygen species.

After BYDV-GAV infection in the susceptible wheat, the lower chlorophyll content, fragmentized chloroplast, poorly-developed thylakoids, rare starch granules and plastoglobules—which were mainly caused by down-regulation of chlorophyll and chloroplast biosynthesis related genes (i.e., *Cab*, *GPT2*, and *GluTR1*)—and ROS excessive accumulation are mainly responsible for the formation of yellowing leaves. The dwarfism formation in the infected wheat plants is mainly attributed to the elevated transcription of ET signaling (*AP2*, and *ERF*) and ABA signaling–related genes (*PLD1* and *CBL9*) and the precisely transcriptional regulation of JA-GA signaling related genes. Based on the above results, we proposed a model to explain major mechanisms underlying the yellow dwarf symptom formation induced by BYDV-GAV infection in susceptible wheat (Figure 6). This research reveals molecular and ultrastructural mechanisms underlying BYDV-induced symptom formation in susceptible wheat.

## Supplementary Materials

Supplementary materials can be found at http://www.mdpi.com/1422-0067/19/4/1187/s1.
