# Supplementary material for: Molecular and Ultrastructural Mechanisms Underlying Yellow Dwarf Symptom Formation in Wheat after Infection of Barley Yellow Dwarf Virus
Source: Int J Mol Sci. 2018 Apr 13;19(4):1187. doi: 10.3390/ijms19041187 (PMC5979330; doi:10.3390/ijms19041187)
Supplement: Supplementary file 1 [file ijms-19-01187-s001.zip › Table S6.docx]

**Table S6 Primers and their sequences used for qRT-PCR experiments**

| **Transcript** | **Putative function** | | **Primers for qRT-PCR** |
| --- | --- | --- | --- |
| Ta.29587.3.A1_at | | Chlorophyll a/b binding protein 1C | F: 5-CGTCAACAACAACGCCTG-3' |
|  |  |  | R: 5'-GCAAAAAGAAACCCAAGC-3' |
| TaAffx.8262.1.S1_x_at | | Glutamyl-tRNA reductase 1 | F: 5'-TCGAGCAGAAGATCAAGG-3' |
|  |  |  | R: 5'-ATTCACGGCACAAGAAGAA-3' |
| Ta.9530.1.S1_at | | Glucose-6-phosphate | F: 5'-ACGGAACAACAAACGAAGA-3' |
|  |  |  | R: 5'-CCAGGCAAAGCAGTAAGG-3' |
| Ta.5198.2.S1_a_at | | GEM-like protein 4 | F: 5'-ATAACACACACAGCGCGTAC-3' |
|  |  |  | R: 5'-AGGCCCAAAACTGTACGAGA-3' |
| Ta.13307.1.S1_x_at | | Peroxidase 9 | F: 5'-TGCTCCCATGTCTCGAACTT-3' |
|  |  |  | R: 5'-ACCCTACCCATTGACCCAAG-3' |
| Ta.8539.3.S1_at | | Calcineurin B-like protein 9 | F：5'-TTGGGACGCTGAGAAACA-3' |
|  |  |  | R：5'-CGTTACAACACATTGGGGA-3' |
| TaAffx.80154.2.S1_at | | Ethylene-responsive transcription factor | F：5'-TAGGTAGGTGGTAGGCAGC-3' |
|  |  |  | R：5'-TACGACCCCAGCCAGATG-3' |
| Ta.21646.1.S1 | | LTPL17 - Protease inhibitor | F: 5'-AGCCATACAGATGCAGTACTACTAT-3' |
|  |  |  | R: 5'-GAGGTAGACTGAGACTTAGCAAAAC-3' |
